# Supplementary figures and images for: Auranofin, identified by FDA-approved drug library screening, inhibits HBs antigen secretion via lysosomal damage
Source: PLoS One. 2026 Jan 16;21(1):e0340023. doi: 10.1371/journal.pone.0340023 (PMC12810816; doi:10.1371/journal.pone.0340023)

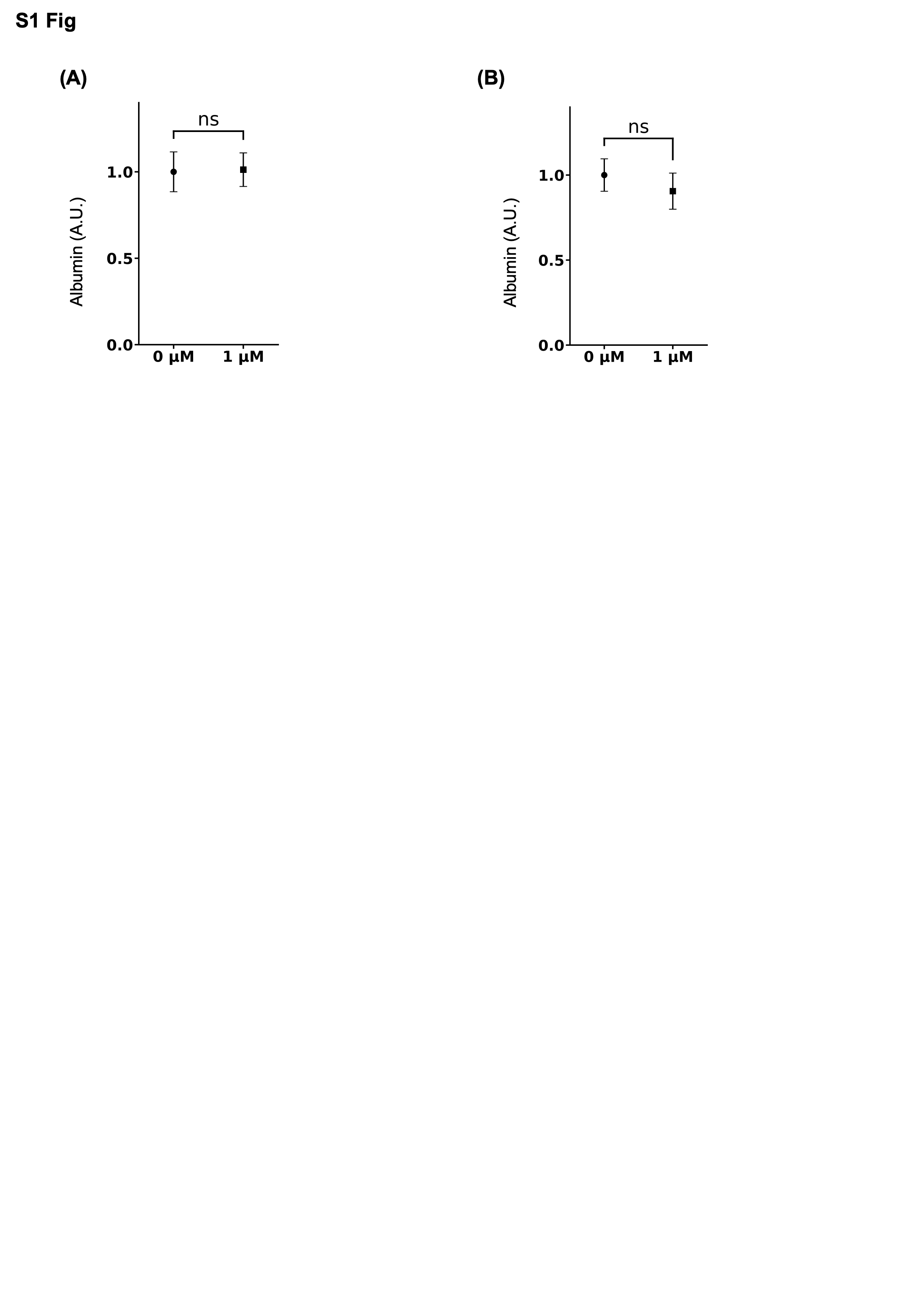

Supplement: S1 Fig — (A) Albumin levels in the supernatant of HepG2.2.15.7 cells treated with or without auranofin. (B) Albumin levels in the supernatant of HBV-infected HepaSH cells treated with or without auranofin. (TIFF) [file pone.0340023.s001.tiff]

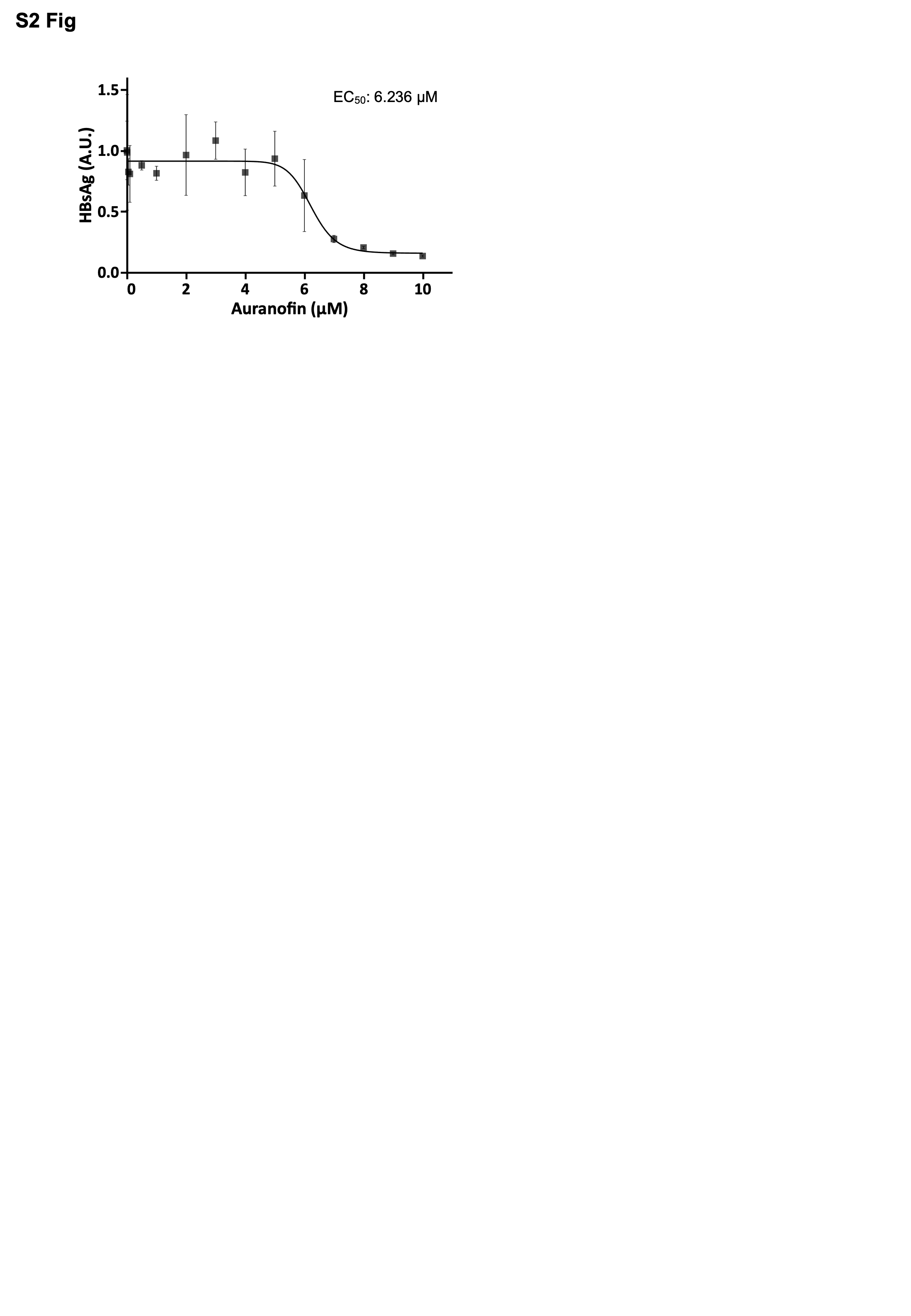

Supplement: S2 Fig — (TIFF) [file pone.0340023.s002.tiff]

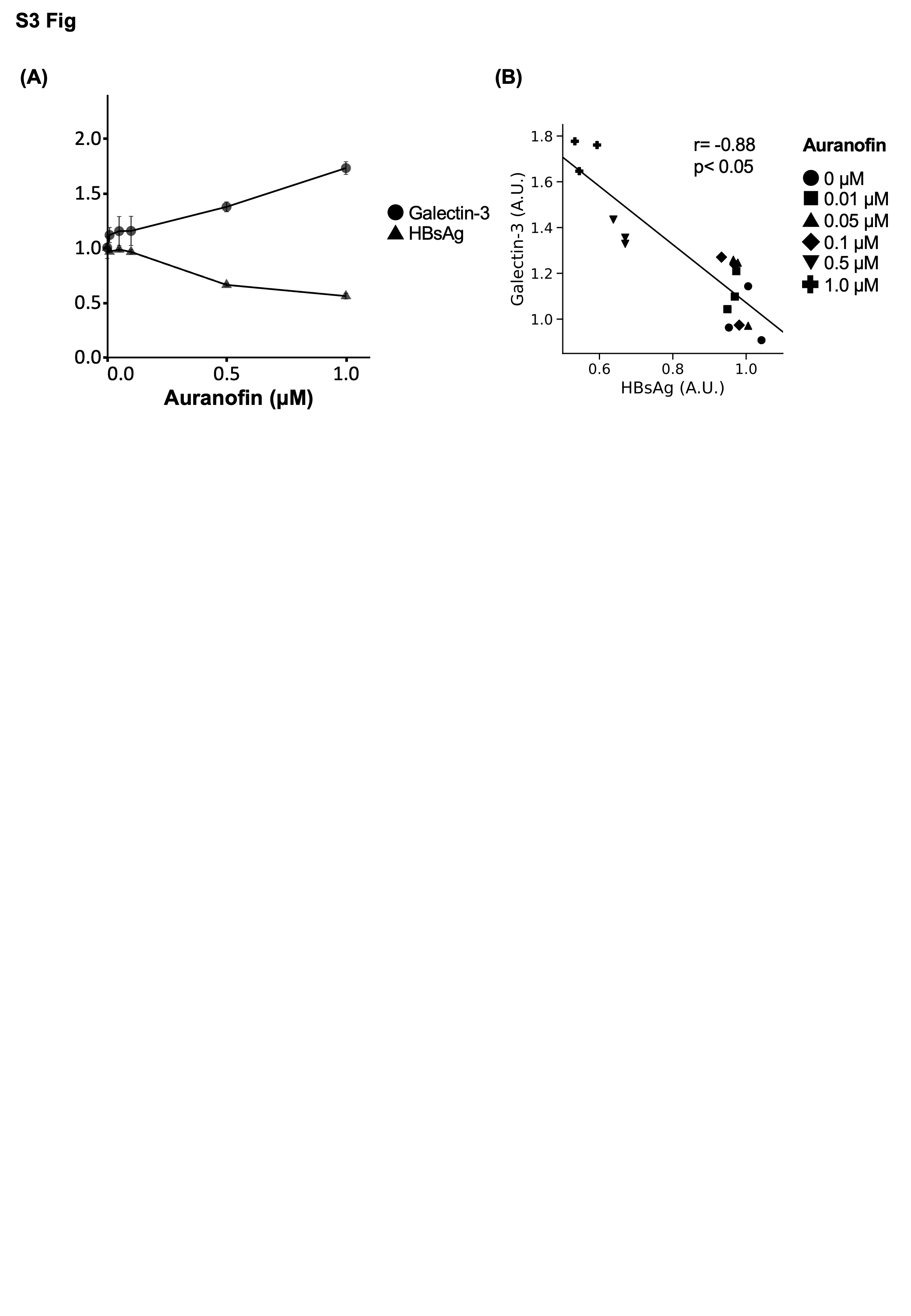

Supplement: S3 Fig — (A) HBsAg levels in the supernatant and mRNA expression levels of Galectin-3 in HepG2.2.15.7 cells treated with various concentrations of auranofin. (B) Correlations between HBsAg levels in the supernatant and Galectin-3 mRNA expression levels in HepG2.2.15.7 cells. (TIFF) [file pone.0340023.s003.tiff]

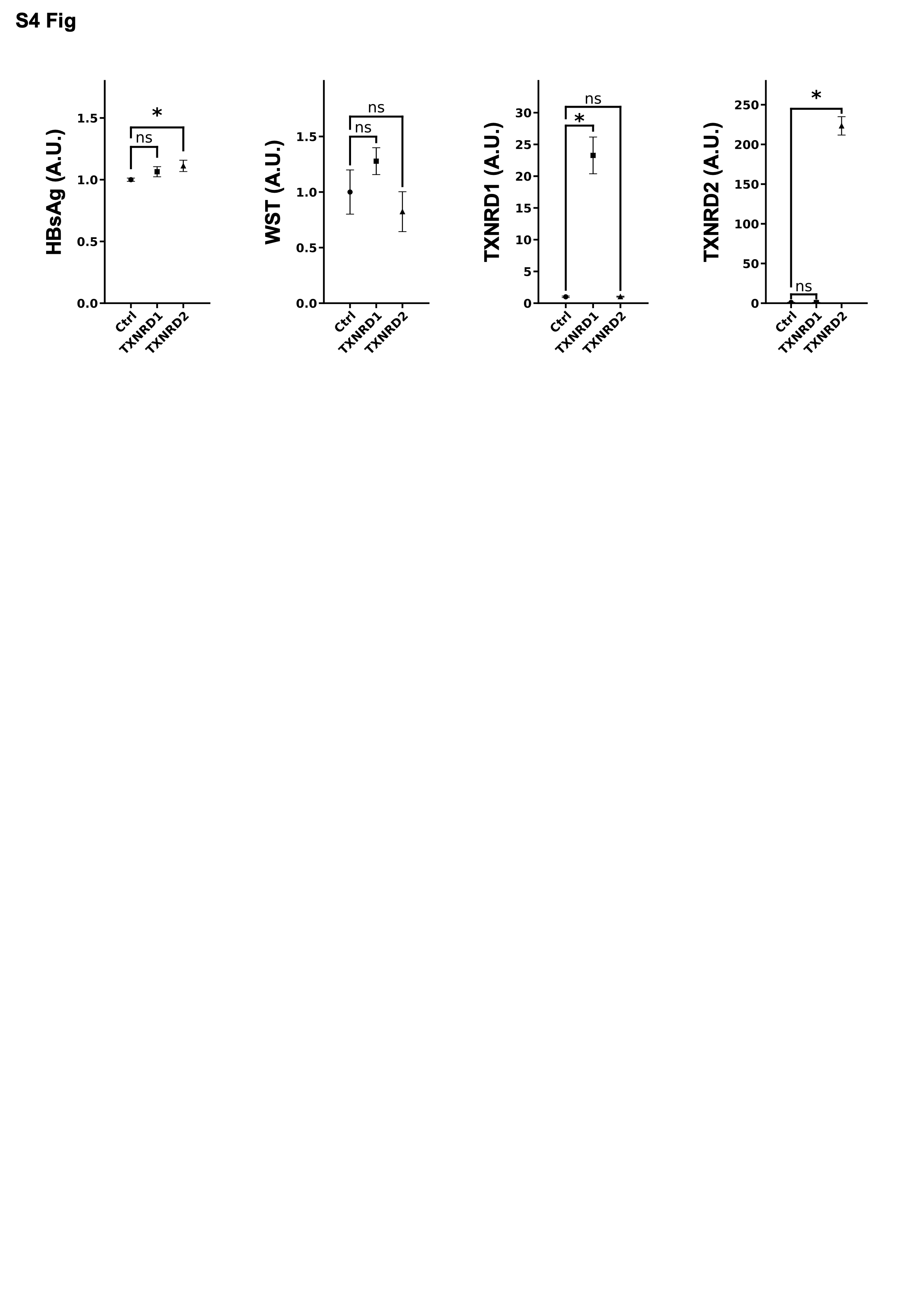

Supplement: S4 Fig — HBsAg levels in the supernatant, cell viability, and TXNRD1/TXNRD2 mRNA expression levels in HepG2.2.15.7 cells with or without TXNRD1 or TXNRD2 overexpression. (*: p < 0.05). (TIFF) [file pone.0340023.s004.tiff]
