# Supplementary material for: Auranofin, identified by FDA-approved drug library screening, inhibits HBs antigen secretion via lysosomal damage
Source: PLoS One. 2026 Jan 16;21(1):e0340023. doi: 10.1371/journal.pone.0340023 (PMC12810816; doi:10.1371/journal.pone.0340023)
Supplement: Attachment — Submitted filename: Response to Reviewers.docx [file pone.0340023.s007.docx]

8 Dec, 2025

Ms. Emily Chenette

Editor-in-Chief, *PLOS One*

Dr. Tatsuo Kanda

Academic Editor, *PLOS One*

Re: Manuscript ID: PONE-D-25-55761

Editors:

Thank you very much for your kind letter. We are grateful for your encouraging review of our manuscript (PONE-D-25-55761), titled “**Auranofin, identified by FDA-approved drug library screening, inhibits HBs antigen secretion via lysosomal damage**”. We have carefully considered your criticisms and comments and have revised our manuscript accordingly.

Point-by-point responses to the comments of the Reviewers are provided below. All changes are highlighted in red.

We hope that the revised manuscript is now suitable for publication in PLOS One.

Sincerely,

Hayato Hikita, M.D., Ph.D.

Department of Gastroenterology and Hepatology,

The University of Osaka Graduate School of Medicine,

2-2 Yamadaoka, Suita, Osaka 565-0871, Japan

Telephone number: +81-6-6879-3621

[hikita@gh.med.osaka-u.ac.jp](mailto:hikita@gh.med.osaka-u.ac.jp)

**Journal Requirements:**

When submitting your revision, we need you to address these additional requirements.

1. Please ensure that your manuscript meets PLOS ONE's style requirements, including those for file naming. The PLOS ONE style templates can be found at

https://journals.plos.org/plosone/s/file?id=wjVg/PLOSOne_formatting_sample_main_body.pdf and

<https://journals.plos.org/plosone/s/file?id=ba62/PLOSOne_formatting_sample_title_authors_affiliations.pdf>

**Reply**

**Thank you for pointing this out. We have thoroughly revised the manuscript and supporting files to ensure full compliance with the PLOS ONE style requirements, including file naming conventions. We have followed the formatting guidelines provided in the links and updated all relevant sections and files accordingly.**

2. We note that the grant information you provided in the ‘Funding Information’ and ‘Financial Disclosure’ sections do not match.

When you resubmit, please ensure that you provide the correct grant numbers for the awards you received for your study in the ‘Funding Information’ section.

**Reply**

**Thank you for bringing this to our attention. We apologize for the inconsistency between the ‘Funding Information’ and ‘Financial Disclosure’ sections. We have carefully reviewed the grant details and corrected the grant numbers in the ‘Funding Information’ section to ensure they accurately reflect the awards that supported this study. The revised sections are now consistent.**

3. Thank you for stating the following financial disclosure:

This study was funded by the Japan Agency for Medical Research and Development (AMED) under grant numbers JP25fk0310534 (K.M., H.H., T.K., and T. Taka.) and JP223fa627006 (Y.H., T. Taka., and H.S.), and by the Basis for Supporting Innovative Drug Discovery and Life Science Research (BINDS).

Please state what role the funders took in the study. If the funders had no role, please state: "The funders had no role in study design, data collection and analysis, decision to publish, or preparation of the manuscript."

If this statement is not correct you must amend it as needed.

Please include this amended Role of Funder statement in your cover letter; we will change the online submission form on your behalf.

**Reply**

**The BINDS provided the FDA-approved drug library used in this study. Aside from providing this material, the funders had no role in study design, data collection and analysis, decision to publish, or preparation of the manuscript.**

4. Thank you for stating the following in the Competing Interests/Financial Disclosure section:

Tetsuo Takehara received research grants from Gilead Sciences, Inc, GSK plc. and Bristol Myers Squibb. Hayato Hikita, Yuki Tahata, and Tetsuo Takehara received lecture fees from Gilead Sciences.

We note that one or more of the authors are employed by commercial companies: Gilead Sciences, Inc, GSK plc. and Bristol Myers Squibb

a. Please provide an amended Funding Statement declaring this commercial affiliation, as well as a statement regarding the Role of Funders in your study. If the funding organization did not play a role in the study design, data collection and analysis, decision to publish, or preparation of the manuscript and only provided financial support in the form of authors' salaries and/or research materials, please review your statements relating to the author contributions, and ensure you have specifically and accurately indicated the role(s) that these authors had in your study. You can update author roles in the Author Contributions section of the online submission form.

Please also include the following statement within your amended Funding Statement.

“The funder provided support in the form of salaries for authors [insert relevant initials], but did not have any additional role in the study design, data collection and analysis, decision to publish, or preparation of the manuscript. The specific roles of these authors are articulated in the ‘author contributions’ section.”

If your commercial affiliation did play a role in your study, please state and explain this role within your updated Funding Statement.

b. Please also provide an updated Competing Interests Statement declaring this commercial affiliation along with any other relevant declarations relating to employment, consultancy, patents, products in development, or marketed products, etc.

Within your Competing Interests Statement, please confirm that this commercial affiliation does not alter your adherence to all PLOS ONE policies on sharing data and materials by including the following statement: "This does not alter our adherence to PLOS ONE policies on sharing data and materials.” (as detailed online in our guide for authors http://journals.plos.org/plosone/s/competing-interests) . If this adherence statement is not accurate and there are restrictions on sharing of data and/or materials, please state these. Please note that we cannot proceed with consideration of your article until this information has been declared.

Please include both an updated Funding Statement and Competing Interests Statement in your cover letter. We will change the online submission form on your behalf.

**Reply**

**Thank you for your comments. None of the authors are employed by Gilead Sciences, Inc., GSK plc., or Bristol Myers Squibb. These companies provided research grants or lecture fees to some authors, as disclosed in the Competing Interests/Financial Disclosure section, but no author receives salary support from these companies. Accordingly, we have amended the Funding Statement and Competing Interests Statement to clarify the sources of funding and the role of funders using the required wording. The updated Funding Statement and Competing Interests Statement is provided below. As requested, we will ensure that the Author Contributions section accurately reflects each author’s roles in the study.**

**Funding Statement:**

**This study was funded by the Japan Agency for Medical Research and Development (AMED) under grant number JP25fk0310534 (K.M., H.H., T.K., and T.Taka.) and JP223fa627006 (Y.H., T.Taka., and H.S.), and Basis for Supporting Innovative Drug Discovery and Life Science Research (BINDS). Tetsuo Takehara received research grants from Gilead Sciences, Inc., GSK plc., and Bristol Myers Squibb. Hayato Hikita, Yuki Tahata, and Tetsuo Takehara received lecture fees from Gilead Sciences. BINDS provided the FDA-approved drug library used in this study. The funders had no role in study design, data collection and analysis, decision to publish, or preparation of the manuscript.**

**Competing Interests Statement:**

**This study was funded by the Japan Agency for Medical Research and Development (AMED) under grant number JP25fk0310534 (K.M., H.H., T.K., and T.Taka.) and JP223fa627006 (Y.H., T.Taka., and H.S.), and Basis for Supporting Innovative Drug Discovery and Life Science Research (BINDS). Tetsuo Takehara received research grants from Gilead Sciences, Inc., GSK plc., and Bristol Myers Squibb. Hayato Hikita, Yuki Tahata, and Tetsuo Takehara received lecture fees from Gilead Sciences. None of the authors are employed by these commercial entities, and the authors declare that they have no other competing interests such as consultancy, patents, products in development, or marketed products. This does not alter our adherence to PLOS ONE policies on sharing data and materials.**

5. Please include your full ethics statement in the ‘Methods’ section of your manuscript file. In your statement, please include the full name of the IRB or ethics committee who approved or waived your study, as well as whether or not you obtained informed written or verbal consent. If consent was waived for your study, please include this information in your statement as well.

**Reply**

**The following statement has been added to the manuscript:**

**“Ethics statement**

**This study did not involve human participants, human data, or human tissue. Therefore, approval by an institutional review board or ethics committee and informed consent were not required. All experimental procedures involving animals were conducted in accordance with the institutional guidelines for animal care and were approved by the Animal Care Committee of the Central Institute for Experimental Medicine and Life Science.”**

6. We note that your Data Availability Statement is currently as follows: All relevant data are within the manuscript and its Supporting Information files.

Please confirm at this time whether or not your submission contains all raw data required to replicate the results of your study. Authors must share the “minimal data set” for their submission. PLOS defines the minimal data set to consist of the data required to replicate all study findings reported in the article, as well as related metadata and methods (https://journals.plos.org/plosone/s/data-availability#loc-minimal-data-set-definition).

For example, authors should submit the following data:

- The values behind the means, standard deviations and other measures reported;

- The values used to build graphs;

- The points extracted from images for analysis.

Authors do not need to submit their entire data set if only a portion of the data was used in the reported study.

If your submission does not contain these data, please either upload them as Supporting Information files or deposit them to a stable, public repository and provide us with the relevant URLs, DOIs, or accession numbers. For a list of recommended repositories, please see https://journals.plos.org/plosone/s/recommended-repositories.

If there are ethical or legal restrictions on sharing a de-identified data set, please explain them in detail (e.g., data contain potentially sensitive information, data are owned by a third-party organization, etc.) and who has imposed them (e.g., an ethics committee). Please also provide contact information for a data access committee, ethics committee, or other institutional body to which data requests may be sent. If data are owned by a third party, please indicate how others may request data access.

**Reply**

**Thank you for your inquiry. We confirm that our submission contains all raw data required to replicate the results of this study. The numerical values underlying all graphs, summary statistics, and quantifications have now been compiled and uploaded as Supporting Information files. These files include the values behind means and standard deviations, the data used to generate graphs, and the quantified data points extracted from images. An updated Data Availability Statement has been added below.**

**Data Availability Statement:**

**All relevant data are within the manuscript and its Supporting Information files. The minimal data set underlying all figures and quantitative analyses, including numerical values used to generate graphs and data points extracted from images, has been provided as Supporting Information.**

7. If the reviewer comments include a recommendation to cite specific previously published works, please review and evaluate these publications to determine whether they are relevant and should be cited. There is no requirement to cite these works unless the editor has indicated otherwise.

**Reply**

**Thank you for your comment. The reviewers did not recommend any additional citations in their comments. We have carefully re-examined the reviewer reports to confirm that no citation requests were made. Therefore, no changes to the References section were required.**

**Review Comments**

Reviewer #1:

The authors focused on auranofin as a potential novel therapeutic agent for HBV infection.

Auranofin failed to decrease intracellular HBsAg or HBV RNA. The authors showed that auranofin only reduced extracellular HBsAg release and increases the number of intracellular Galectin-3–positive vesicles in HBV-infected cells. Based on these findings, the authors concluded—and reflected in the title—that lysosomal damage is responsible for the reduced extracellular release of HBsAg. Since the major novelty of this study lies in the finding that auranofin is associated with reduced HBsAg release, it would be essential to elucidate the molecular mechanism underlying this effect. Without mechanistic evidence linking lysosomal damage directly to impaired HBsAg secretion, publication of this work would be difficult to justify.

**Reply**

**We sincerely thank the reviewer for the insightful and constructive comments. We agree that elucidating the mechanistic link between auranofin-induced lysosomal damage and the reduction of extracellular HBsAg release is crucial for strengthening the novelty and significance of our study. In response to the reviewer’s comment, we have now conducted additional experiments to further clarify the underlying mechanism.**

#1. While the increase in Galectin-3 and damaged lysosomes after auranofin treatment in HBV-infected HepaSH cells suggests lysosomal injury, this observation alone does not directly demonstrate that lysosomal damage contributes to the reduced extracellular release of HBsAg.The authors could perform rescue experiments using antioxidants or lysosomal membrane–stabilizing interventions to determine whether preventing lysosomal damage restores HBsAg secretion under Auranofin treatment.

**Reply**

**- Thank you for the important suggestion. As the reviewer noted, we did not directly demonstrate that the increase in Galectin-3** **contributes to the reduced extracellular release of HBsAg.** **Autophagy induced following lysosomal damage caused by B5, a thioredoxin reductase inhibitor similar to auranofin, has been reported to occur in a ROS-independent manner (Shao FY, et al. B5, a thioredoxin reductase inhibitor, induces apoptosis in human cervical cancer cells by suppressing the thioredoxin system, disrupting mitochondrion-dependent pathways and triggering autophagy. Oncotarget. 2015 Oct 13;6(31):30939-56. doi: 10.18632/oncotarget.5132.), and thus cannot be effectively rescued by antioxidants. We therefore examined the effect of overexpressing thioredoxin reductase—the known target inhibited by auranofin—on extracellular HBsAg levels.**

**In HepG2.2.15.7 cells, the thioredoxin reductases TXNRD1 and TXNRD2 were overexpressed. Compared with the control group, the group in which TXNRD1 was overexpressed did not have altered HBsAg levels in the culture supernatant, whereas the group in which TXNRD2 was overexpressed had increased extracellular HBsAg levels. We added these data to S4 Fig, and rewrote the Results section (p. 21 l 385).**

**S4 TXNRD2 overexpression resulted in an increase in extracellular HBsAg levels.** HBsAg levels in the supernatant, cell viability, and TXNRD1/TXNRD2 mRNA expression levels in HepG2.2.15.7 cells with or without TXNRD1 or TXNRD2 overexpression. (*: p<0.05).

**Although TXNRD2 overexpression partially restored extracellular HBsAg release, we acknowledge that this approach may not directly demonstrate a causal relationship between lysosomal injury and impaired HBsAg secretion. To clarify this mechanistic link more conclusively, further studies aimed at directly modulating lysosomal integrity are needed, and this limitation has been noted in the Discussion (p. 23 l 440).**

#2. The correlation between the degree of Galectin-3 expression or the number of Galectin-3–positive vesicles with the amount of extracellular HBsAg would strengthen the conclusion. Although no clear dose-dependency of auranofin was observed, this point is somewhat concerning, as it raises questions about the reproducibility and mechanism of the observed effects. A dose–response or time-course analysis showing that lysosomal damage (as indicated by Galectin-3 puncta) quantitatively associates with decreased HBsAg release would provide more convincing mechanistic evidence.

**Reply**

**- Thank you for the important suggestion. As the reviewer noted, demonstrating that lysosomal damage quantitatively correlates with decreased HBsAg release is critically important for providing convincing mechanistic evidence.**

**Treatment of HepG2.2.15.7 cells with auranofin led to a dose-dependent increase in Galectin-3 mRNA expression. In addition, Galectin-3 mRNA expression levels were negatively correlated with HBsAg levels in the culture supernatant. We added these data to S3 Fig, and rewrote the Results section (p. 20 l 380).**

**S3 Fig. Treatment of HepG2.2.15.7 cells with auranofin led to a dose-dependent increase in Galectin-3 mRNA expression, and Galectin-3 mRNA expression levels were negatively correlated with HBsAg levels in the culture supernatant.** (A) HBsAg levels in the supernatant and mRNA expression levels of Galectin-3 in HepG2.2.15.7 cells treated with various concentrations of auranofin. (B) Correlations between HBsAg levels in the supernatant and mRNA expression levels of Galectin-3 in HepG2.2.15.7 cells.

Reviewer #2:

This is a well-designed repositioning study that identifies auranofin as a reducer of HBsAg via inhibition of extracellular release. The experimental design and mechanistic dissection are coherent, and the findings are virologically insightful. I recommend publication after the following minor points are addressed:

**Reply**

**We appreciate your time and effort in reviewing our manuscript. We are also very grateful to you for noting the importance of our work.**

1. Please report the EC50 for auranofin. If possible, ideally include values for both models (HepG2.2.15.7 and primary human hepatocytes).

**Reply**

**- Thank you for the important suggestion. As the reviewer noted,** **it is important to determine the EC_50_ of auranofin in the models used in the present study (HepG2.2.15.7 cells and primary human hepatocytes). The EC₅₀ of auranofin in HepG2.2.15.7 cells was 0.375 μM.** **The EC_50_ of auranofin in HepaSH cells was 6.236 μM. We added these data to Figure 2A and Supplementary Figure 1C, and rewrote the Results section (p. 18 l 322 and l 332).**

2. Please assess whether auranofin affects host-protein secretion (e.g., ALB).

**Reply**

**- Thank you for the important suggestion. Evaluating whether auranofin inhibits not only HBsAg secretion but also host protein secretion is critically important from the perspective of developing novel therapeutic agents against HBV. In HBV-infected HepaSH cells and HepG2.2.15.7 cells, auranofin treatment did not reduce albumin levels in the culture supernatant. We added these data to Supplementary Figure 1A and B, and rewrote the Results section (p. 18 l 330).**

3. Please unify terminology (“HBsAg”).

The manuscript alternates between “HBs antigen” and “HBsAg.”

**Reply**

**- Thank you very much for your thorough review of our manuscript. We have standardized the terminology throughout the manuscript by consistently using “HBsAg.”**
